# Idade Avançada Reduz a Tipicidade da Apresentação Clínica em Pacientes Com Dor Torácica Aguda Relacionada a Doença Coronária Obstrutiva?

**DOI:** 10.36660/abc.20190089

**Published:** 2021-06-08

**Authors:** Pedro Henrique Correia Filgueiras, Antônio Maurício Cerqueira, Gabriela Oliveira Bagano, Vitor Calixto de Almeida Correia, Fernanda Oliveira de Andrade Lopes, Thiago Menezes Barbosa de Souza, Leticia Lara Fonseca, Lara Queiroz Kertzman, Yasmin Falcon Lacerda, Marcia Noya Rabelo, Luis Claudio Lemos Correia

**Affiliations:** 1 Escola Bahiana de Medicina e Saúde Pública SalvadorBA Brasil Escola Bahiana de Medicina e Saúde Pública (EBMSP), Salvador , BA - Brasil; 2 Hospital São Rafael SalvadorBA Brasil Hospital São Rafael , Salvador , BA - Brasil

**Keywords:** Idoso, Dor no Peito, Síndrome Coronariana Aguda, Prognóstico

## Abstract

**Fundamento:**

De acordo com o pensamento diagnóstico tradicional, indivíduos muito idosos estão mais predispostos a desenvolver sintomas atípicos em síndromes coronarianas agudas.

**Objetivo:**

Testar a hipótese de que indivíduos muito idosos estão mais predispostos a manifestações de dor torácica atípica devido à doença arterial coronariana obstrutiva (DAC).

**Métodos:**

O Registro de dor torácica inclui pacientes internados com dor torácica aguda. Primeiramente, foi construído o índice de tipicidade dessa manifestação clínica: a soma de 12 características de sintomas (8 sintomas típicos e 4 sintomas atípicos). No subgrupo de pacientes com etiologia coronariana, o índice de tipicidade foi comparado entre octogenários e não octogenários. A significância estatística foi definida por p<0,05.

**Resultados:**

958 pacientes foram incluídos no registro, sendo que 486 (51%) tinham etiologia supostamente coronariana. Nesse grupo, 59 (12%) octogenários (idade 84±3,5; 50% homens) foram comparados a 427 pacientes com idade <80 (60±12 anos; 71% homens). O índice de tipicidade em octogenários foi 3,42±1,92, que é semelhante ao de não octogenários (3,44±1,74; p=0,092 na análise univariada e p=0,80 após ajuste para sexo pela análise de variância — ANOVA). Também não houve diferença estatisticamente significativa quando a amostra foi dividida em idade mediana (62 anos; 3,41±1,77 vs. 3,49 ± 1,77; p=0,61). Não houve associação linear estatisticamente significativa entre idade e índice de tipicidade (r=- 0,05; p=0,24). A análise de regressão logística para predição de DAC na amostra geral de 958 pacientes não mostrou interação do índice de tipicidade com a idade numérica (p=0,94), octogenários (p=0,22) ou idade acima da mediana (p=0,74).

**Conclusão:**

Em pacientes com dor torácica aguda de etiologia coronariana, a idade avançada não influencia o quadro clínico típico.

## Introdução

O pensamento clínico tradicional indica que os idosos estão predispostos a sintomas atípicos nas síndromes coronarianas agudas (SCA), condição que pode implicar em difícil diagnóstico e tratamento tardio. ^1^ Os mecanismos plausíveis para atipicidade seriam limitações cognitivas, comunicação comprometida ou redução da percepção da dor. ^2^

No entanto, embora esse pensamento clínico tradicional tenha como possível base fisiológica a alteração nociceptiva causada pela depressão e diabetes, que são mais prevalentes em indivíduos com mais idade, a grande maioria dos estudos encontrados na literatura é retrospectiva e com uma definição bastante variável e subjetiva de “tipicidade da dor”. Portanto, ainda não está claro se, de fato, a idade avançada implica um quadro clínico diferente no contexto das síndromes coronarianas. ^3 , 4^

Assim, o presente estudo se propõe a testar a hipótese de que indivíduos muito idosos estão mais predispostos a manifestações atípicas de dor torácica de etiologia coronariana. Como uma análise primária, a tipicidade geral da manifestação clínica foi comparada entre octogenários e não octogenários na subamostra de pacientes com etiologia coronariana. Seguiu-se a análise da interação entre idade e tipicidade da dor na predição da etiologia coronariana na amostra de todas as etiologias de dor torácica.

## Métodos

### Seleção da amostra

O Registro de Dor Torácica é uma amostra de pacientes internados consecutivamente na Unidade Coronariana de um hospital terciário de setembro de 2011 a dezembro de 2017, principalmente por desconforto torácico, independentemente de alterações eletrocardiográficas, marcadores de necrose ou qualquer outro exame complementar que mostre a causa do sintoma.

A amostra selecionada tem como objetivo representar a população-alvo de pacientes internados na unidade coronariana devido a dor torácica. Assim, todos os pacientes internados durante o período do estudo foram incluídos no estudo, não havendo seleção de subamostra nesta população. A internação na unidade coronariana não foi influenciada pelo protocolo do estudo. A probabilidade diagnóstica foi estabelecida a critério dos médicos assistentes.

O estudo está de acordo com as normas éticas da resolução 510/2016 do Ministério da Saúde, foi aprovado pelo Comitê de Ética em Pesquisa Hospitalar e todos os participantes assinaram o termo de consentimento livre e esclarecido.

### Caracterização do desconforto torácico

Na internação, as informações sobre a apresentação clínica do desconforto torácico foram coletadas por meio de entrevista parametrizada. A entrevista foi realizada de forma sistematizada por pesquisadores treinados para evitar induzir as respostas dos pacientes e focar na reprodutibilidade do método. A entrevista foi parametrizada para exigir respostas objetivas sim/não. Quando o paciente manifestou dúvida, o sintoma foi considerado ausente.

Foram avaliadas 12 características de sintomas, incluindo 8 características consideradas típicas de angina (dor precordial, aspecto compressivo, irradiação para membro superior esquerdo, irradiação para o pescoço, intensidade classificada pelo paciente como grave, desconforto nos dias anteriores, presença de sintomas vagais, administração de medicação sublingual seguida de melhora do sintoma) e 4 características consideradas atípicas (mudança da dor conforme a posição, mudança com palpação do local, mudança com movimento do braço e mudança com respiração).

### Índice de tipicidade de sintomas

Para quantificar a tipicidade geral da manifestação clínica, atribuiu-se 1 ponto a cada característica típica e subtraiu-se 1 ponto para cada característica atípica (variação de -4 a +8, proporcional à tipicidade).

### Definição da etiologia dos sintomas

Para a avaliação diagnóstica, os pacientes foram submetidos a angiografia coronária invasiva ou teste provocativo não invasivo (ressonância magnética de perfusão nuclear e tomografia computadorizada por emissão de fóton único ou ecocardiografia sob estresse com dobutamina), a critério do cardiologista responsável. Para testes não invasivos positivos, os pacientes fizeram uma angiografia para confirmação. Com base nesse algoritmo de diagnóstico, definiu-se doença arterial coronariana obstrutiva (DACO) como estenose ≥70% na angiografia. A angiografia coronária sem lesão obstrutiva ou teste não invasivo normal (tamanho do defeito isquêmico <5% do miocárdio ventricular esquerdo) indicou ausência de DACO.

### Análise dos dados

A normalidade das variáveis numéricas foi testada por histograma, comparando média e mediana, e considerando principalmente o nível de curtose e assimetria <3. Indivíduos muito idosos foram definidos com idade ≥80 anos (octogenários). Realizou-se a análise preliminar na amostra de pacientes com doença coronariana obstrutiva, comparando o índice de tipicidade entre octogenários e não octogenários. Além disso, comparou-se cada característica de sintomas entre os dois grupos. As variáveis numéricas foram expressas como média e desvio padrão, comparadas entre os dois grupos pelo teste t de Student não pareado. As variáveis categóricas foram expressas em proporções e comparadas com o teste qui-quadrado de Pearson. Procedeu-se à análise de variância para comparar o índice de tipicidade entre os grupos após ajuste para sexo. A associação linear entre índice de tipicidade e idade foi testada pelo coeficiente de correlação de Pearson, com base na distribuição normal de ambas as variáveis. A comparação múltipla foi ajustada pelo método de Bonferroni.

Em seguida, utilizou-se a amostra total do registro (todos os pacientes admitidos com dor torácica aguda, com e sem doença arterial coronariana), e avaliamos a capacidade preditiva do índice de tipicidade para doença arterial coronariana obstrutiva com base na área sob a curva *Receiver Operator Characteristic* (ROC). Depois, avaliamos o efeito modificador da idade sobre a precisão do diagnóstico (DACO) da tipicidade geral da dor, em termos de interação vs. tipicidade da idade na regressão logística, com a idade sendo inserida de três maneiras diferentes: como uma variável numérica, categorizada em dois grupos (octogenários ou não octogenários) e categorizada em dois grupos a partir da mediana da amostra. Para a análise estatística, foi utilizado o software SPSS versão 23. A significância estatística foi definida como um valor de p bicaudal menor que 0,05.

### Cálculo amostral

Quanto ao cálculo amostral, trata-se de um estudo realizado em amostra previamente existente no Registro de Dor Torácica, uma coleta prospectiva de pacientes internados por dor torácica. Esse registro é utilizado para várias análises e, em nossa metodologia, antes de decidirmos testar qualquer hipótese, avaliamos o poder estatístico, que depende do comportamento da variável em questão. Assim, como os dados já haviam sido coletados, poderíamos utilizar o desvio padrão da amostra que seria utilizado para avaliar se o tamanho da amostra tinha poder suficiente, critério essencial para permitir a análise dos dados em nosso protocolo.

Assim, o tamanho da amostra foi definido primeiro, com base na distribuição do índice de tipicidade na amostra de doença coronariana. Considerando o desvio padrão de 1,7, seria necessário que 36 octogenários e 109 não octogenários oferecessem 80% de poder na detecção de uma diferença de 30% no índice típico pelo teste t de Student.

## Resultados

### Caracterização da amostra

Entre setembro de 2011 e dezembro de 2017, 958 indivíduos foram incluídos no registro, e 486 (51%) tinham etiologia supostamente coronariana. Nesse grupo, 59 octogenários foram comparados a 427 não octogenários. A média de idade dos octogenários foi de 85±3,4 anos, sendo 56% homens, em comparação com 60±12 anos, sendo 71% homens, no grupo de não octogenários (p<0,001). Pacientes octogenários tiveram uma prevalência maior de disfunção ventricular esquerda clinicamente manifesta (24% versus 8,7%, p<0,001), doença triarterial ou tronco de coronária esquerda (41% versus 26%, p=0,01) e menor prevalência de infarto agudo do miocárdio com elevação do segmento ST (25% versus 30%, p<0,001). A mortalidade foi maior no grupo com idade mais avançada (14% versus 2,1%, p<0,001). As variáveis comparadas entre os dois grupos encontram-se discriminadas na tabela 1 .

**Tabela 1:** – Características clínicas e comorbidades

	Idade <80 anos (n=427)	Idade ≥80 anos (n=59)	Valor de p
Idade (anos)	60±12	85±3,4	<0,001
Homens	302 (71%)	33 (56%)	0,02
Isquemia ao ECG	279 (67%)	37 (65%)	0,80
Troponina positiva	274 (65%)	49 (83%)	0,005
Infarto com supradesnivelamento do segmento ST	127 (30%)	15 (25%)	<0,001
Diabetes mellitus	161 (38%)	27 (46%)	0,23
Creatinina (mg/dL)	1,0±0,69	1,1±0,43	0,12
Pressão arterial sistólica (mmHg)	154±31	153±36	0,08
Frequência cardíaca (bpm)	78±18	76±19	0,17
Doença coronariana prévia	139 (33%)	24 (41%)	0,22
Revascularização miocárdica prévia	37 (8,7%)	7 (12%)	0,40
Padrão anatômico grave*	80 (26%)	16 (41%)	0,01

*Cateterismo com obstrução ≥70%. ECG: eletrocardiograma.

### Idade e tipicidade dos sintomas

**Figura 1:**
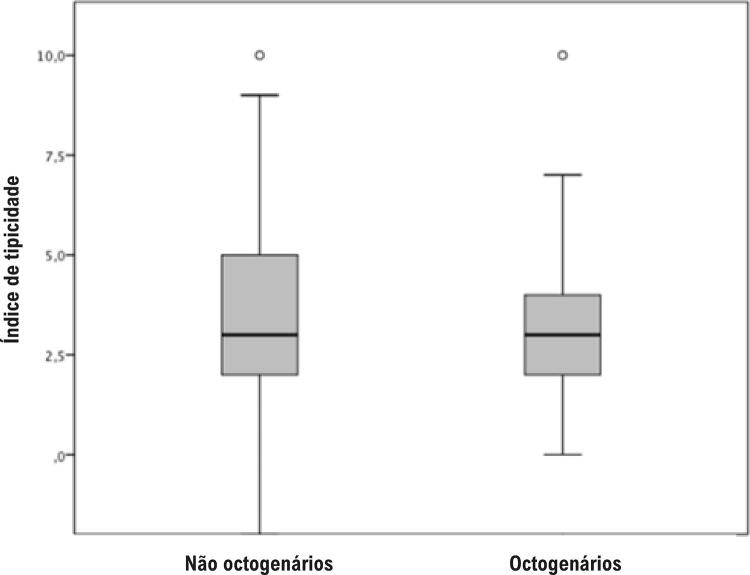
– Boxplot do índice de tipicidade para os grupos octogenários e não octogenários (p=0,92).

O índice de tipicidade dos pacientes muito idosos foi de 3,42±1,92, semelhante ao observado em indivíduos mais jovens (3,44±1,74; p=0,92). A comparação do índice de tipicidade permaneceu não significativa (p=0,80) após ajuste para diferença de gênero entre os grupos ( Figura 1 ).

**Figura 2:**
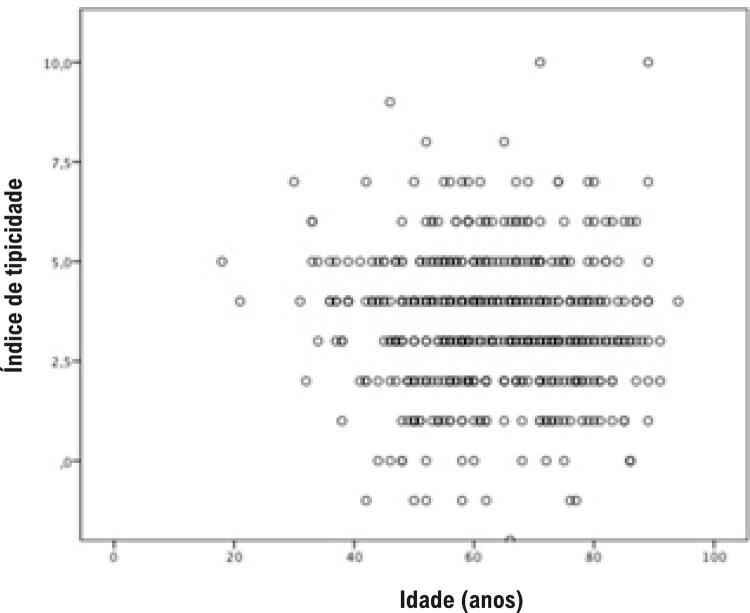
– Gráfico de dispersão do índice típico e idade.

Não houve diferença no índice de tipicidade quando a amostra foi dividida em idade mediana (62 anos), sendo 3,41±1,77 versus 3,49±1,77 (p=0,61). Da mesma forma, não houve correlação entre índice típico e idade (r=- 0,05, p=0,24) ( Figura 2 ).

A comparação entre as 12 características da dor entre octogenários e não octogenários não mostrou diferença significativa após o ajuste de Bonferroni ( Tabela 2 ).

**Tabela 2:** – Características da dor torácica

	Idade ≥80 anos	Idade <80 anos	Valor de p	Valor de p ajustado (Bonferroni)
Dor precordial	348 (82%)	48 (81%)	0,98	--
Dor compressiva	241 (56%)	28 (48%)	0,19	--
Irradiação para o membro superior esquerdo	167 (39%)	19 (32%)	0,02	0,24
Irradiação para o pescoço	110 (26%)	8 (14%)	0,04	0,48
Intensidade severa	253 (60%)	39 (66%)	0,33	--
Desconforto nos dias anteriores	67 (48%)	14 (67%)	0,10	--
Sintomas vagais	215 (50%)	32 (54%)	0,58	--
Melhora com nitrato	182 (43%)	18 (31%)	0,08	--
Muda com a posição	70 (16%)	7 (12%)	0,37	--
Muda com palpação	26 (6,1%)	2 (3,4%)	0,40	--
Muda com o movimento do braço	29 (6,8%)	3 (5,1%)	0,62	--
Dor pleurítica	51 (12%)	5 (8,5%)	0,43	--

### Efeito modificador da idade na capacidade preditiva da tipicidade da dor

**Figura 3:**
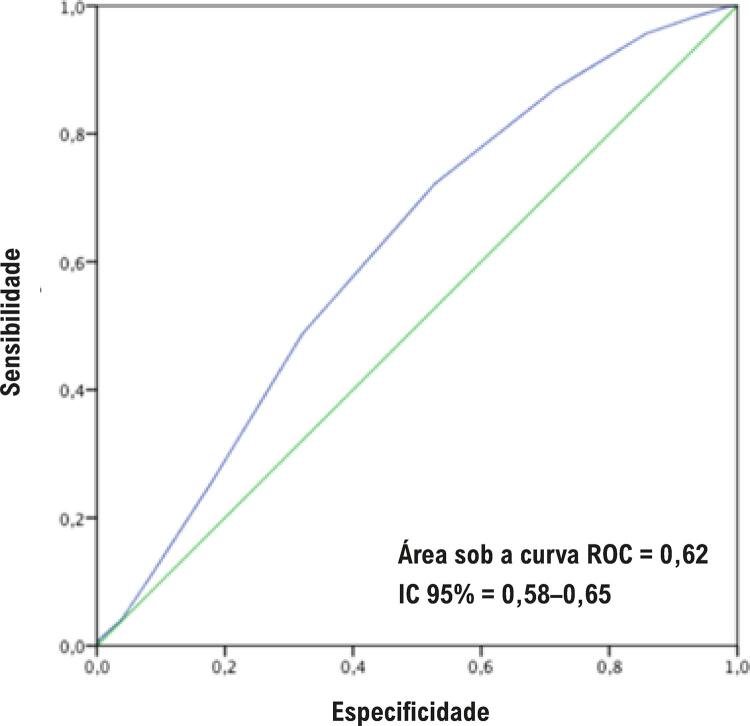
– Estatística C do índice de tipicidade para predição de doença arterial coronariana obstrutiva, considerando todos os pacientes no registro (958), curva ROC: 0,62 (95% CI = 0,58–0,65).

Analisando os 958 pacientes do registro, o índice de tipicidade apresentou uma área sob a curva ROC de 0,62 (IC 95% = 0,58–0,65) para predição de doença arterial coronariana obstrutiva. A análise de regressão logística não demonstrou interação do índice de tipicidade com idade numérica (p=0,94), octogenários (p= 0,22) ou idade acima da mediana de 62 anos (p=0,74) ( Figura 3 ).

## Discussão

O presente estudo demonstra que a idade avançada não influencia a tipicidade da apresentação clínica no contexto das síndromes coronarianas agudas. Além disso, o valor diagnóstico da manifestação clínica não é influenciado pela idade. Conforme mostrado, mesmo analisando a “idade avançada” sob várias perspectivas (dividindo a amostra entre octogenários e não octogenários, idade mediana, 62 anos, e ainda colocando a idade como uma variável contínua), nenhuma das análises sugeriu influência.

A utilização de um “índice de tipicidade” permitiu analisar a tipologia geral da dor, informação complementada pela análise individual de cada característica. O chamado “índice” é apenas a contagem dos sintomas sugeridos menos os sintomas não sugeridos, uma forma de tratar a tipicidade como variável numérica, evitando a subjetividade da categorização em um quadro clínico típico ou atípico.

Outro ponto importante deste estudo é que, para a definição de DACO, utilizamos a cinecoronariografia, exame padrão-ouro, o que implica baixo risco de viés de calibração.

Estudos anteriores que buscaram estudar a dor em indivíduos com idade mais avançada com síndrome coronariana aguda mostraram resultados controversos. ^2 , 3 , 5^ Verifica-se que na maioria desses estudos a coleta das características da dor foi feita retrospectivamente e a partir de bancos de dados desenvolvidos com outros objetivos primários.

Em 2001, Mehta et al., ^1^ por meio de um registro de beneficiários do Medicare nos EUA, selecionaram pacientes com diagnóstico de infarto agudo do miocárdio e estratificaram a amostra com base na idade. ^1^ Em seu estudo, os autores concluem que a apresentação inicial da dor torácica diminui com o aumento da idade. No entanto, não mostram se há diferença estatística entre os valores, o que torna essa conclusão enganosa.

Em uma análise post-hoc do registro *Internet Tracking Registry for Acute Coronary Syndromes* (i*trACS), Han et al., ^3^ analisaram a apresentação clínica em pacientes com SCA de dois grupos: idade ≥75 anos e <75 anos. Classificaram a “apresentação típica” como dor torácica em esmagamento, compressão ou pressão e concluíram que apenas no grupo de pacientes mais jovens (idade <75 anos) a apresentação típica estava associada ao diagnóstico de SCA. Além da definição simplista de “apresentação típica”, os autores não compararam as duas faixas etárias com diagnóstico de SCA. Em outra análise post-hoc, do *Gulf Registry of Acute Coronary Events* ( *Gulf RACE* ), El-Menyar et al. ^5^ classificaram em 3 categorias: típica, atípica e dispneia, não sendo encontrada diferença de idade nos grupos de apresentação “típica” (55±12) e “atípica” (57±13). No entanto, os autores atribuem características bastante amplas como sendo “típicas”: “irradiação para o braço, ombro, costas, pescoço, mandíbula, epigástrio ou outros locais,” o que torna essa classificação subjetiva. Devemos reconhecer que, apesar de satisfazer o cálculo amostral, nossa população de pacientes muito idosos era pequena. Além disso, nosso estudo foi realizado em apenas um centro e em uma população selecionada, sendo necessário o desenvolvimento de novos estudos neste contexto. Reconhecemos também que este estudo foi realizado em ambiente de hospital terciário, portanto, devemos ter cuidado ao extrapolar seus resultados para o ambiente de atenção primária. Nossa população de maior interesse é a de pacientes internados em unidade coronariana, população em que o desafio da discriminação diagnóstica é maior, pois há maior homogeneidade de sintomas (zona de probabilidade cinza). Por ser nossa população-alvo, não houve viés de seleção. Finalmente, existe uma infinidade de possibilidades e combinações de sintomas a serem incluídos em uma análise como esta. Mas aqui, não estamos tentando criar um escore preditor para a etiologia da dor; estamos apenas comparando os muito idosos com os não muito idosos quanto à “carga de tipicidade”. Independentemente de contemplarmos todos os sintomas possíveis, o teste de hipótese para a “carga de tipicidade” não fica comprometido. Estamos apenas avaliando se existe um gradiente de sintomas entre esses dois grupos.

## Conclusão

Em pacientes com dor torácica de etiologia coronariana, a idade avançada não parece influenciar a apresentação clínica típica, sugerindo que os sintomas devam ser interpretados independentemente da idade.
