# Supplementary material for: Idade Avançada Reduz a Tipicidade da Apresentação Clínica em Pacientes Com Dor Torácica Aguda Relacionada a Doença Coronária Obstrutiva?
Source: Arq Bras Cardiol. 2021 Jun 8;116(6):1039–45. [Article in Portuguese] doi: 10.36660/abc.20190089 (PMC8288541; doi:10.36660/abc.20190089)
Supplement: Translation

# Translation: Does Advanced Age Reduce the Typicality of Clinical Presentation in Patients with Acute Chest Pain Related to Coronary Artery Disease?

**Authors:** Pedro Henrique Correia Filgueiras, Antônio Maurício Cerqueira, Gabriela Oliveira Bagano, Vitor Calixto de Almeida Correia, Fernanda Oliveira de Andrade Lopes, Thiago Menezes Barbosa de Souza, Leticia Lara Fonseca, Lara Queiroz Kertzman, Yasmin Falcon Lacerda, Marcia Noya Rabelo, Luis Claudio Lemos Correia

**Affiliations:** 1 Brazil Escola Bahiana de Medicina e Saúde Pública (EBMSP), Salvador, BA - Brazil; 2 Brazil Hospital São Rafael, Salvador, BA - Brazil

**Keywords:** Elderly, Chest Pain, Acute Coronary Syndrome, Prognosis

## Abstract

**Background:**

According to traditional diagnosis thinking, very elderly individuals are more predisposed to develop atypical symptoms in acute coronary syndromes.

**Objective:**

To test the hypothesis that very elderly individuals are more predisposed to atypical chest pain manifestations due to obstructive coronary artery disease (CAD).

**Methods:**

The Registry of Thoracic Pain includes patients admitted with acute chest pain. Firstly, the typicality index of this clinical manifestation was constructed: the sum of 12 symptom characteristics (8 typical and 4 atypical symptoms). In the subgroup of patients with coronary etiology, the typicality index was compared between octogenarian and non-octogenarian individuals. Statistical significance was defined by p<0.05.

**Results:**

958 patients were included in the registry, and 486 (51%) had a supposedly coronary etiology. In this group, 59 (12%) octogenarians (age 84±3.5, 50% men) were compared to 427 patients aged <80 (60±12 years, 71% men). The typicality index in octogenarians was 3.42±1.92, which is similar to that of non-octogenarians (3.44±1.74; p=0.92 in univariate analysis and p=0.80 after adjustment for sex by analysis of variance — ANOVA). There was also no statistically significant difference when the sample was divided into median age (62 years; 3.41±1.77 vs. 3.49 ± 1.77; p=0.61). There was no statistically significant linear association between age and typicality index (r=- 0.05; p=0.24). Logistic regression analysis for prediction of CAD in the general sample of 958 patients showed no interaction of typicality index with numeric age (p=0.94), octogenarians (p=0.22) or age above median (p=0.74).

**Conclusion:**

In patients with acute chest pain of coronary etiology, advanced age does not influence the typical clinical presentation.

## Introduction

Traditional clinical thinking indicates that elderly individuals are predisposed to atypical symptoms in acute coronary syndromes (ACS), a condition that may imply difficult diagnosis and delayed treatment.^1^ The plausible mechanisms for atypicality would be cognitive limitations, compromised communication or reduction of pain perception.^2^

However, although this traditional clinical thinking has as a possible physiological basis nociceptive alteration caused by depression and diabetes, which are more prevalent in older individuals, the vast majority of the studies found in the literature are retrospective and with a fairly variable and subjective definition of “pain typicality”. Therefore, it is still not clear whether, in fact, old age implies a different clinical presentation in the context of coronary syndromes.^3 , 4^

Thus, the present study proposes to test the hypothesis that very elderly individuals are more predisposed to atypical manifestations of chest pain of coronary etiology. As a primary analysis, the overall typicality of the clinical manifestation was compared between octogenarians and non-octogenarians in the subsample of patients with coronary etiology. This was followed by the analysis of the interaction between age and pain typicality in the prediction of coronary etiology in the sample of all etiologies of thoracic pain.

## Methods

### Sample selection

The Registry of Chest Pain is a sample of patients consecutively admitted to the Coronary Unit of a tertiary hospital from September 2011 to December 2017, primarily for chest discomfort, regardless of electrocardiographic abnormalities, necrosis markers or any other complementary examination showing the cause of the symptom.

The selected sample aims to represent the target population of patients admitted to the coronary unit due to chest pain. Thus, all patients admitted during the study period were included in the study, with no subsample selection in this population. Admission to the coronary care unit was not influenced by the study protocol. The diagnostic probability was established at the discretion of the attending physicians.

The study is in accordance with the ethical standards of resolution 510/2016 from the Ministry of Health, was approved by the Committee of Ethics in Hospital Research, and all subjects signed a free and informed consent.

### Characterization of thoracic discomfort

Upon admission, information about the clinical presentation of chest discomfort was collected through a parameterized interview. This interview was done in a systematized way by trained investigators to avoid inducing patients’ responses and to focus on the reproducibility of the method. The interview was parameterized to require objective yes/no answers. When the patient expressed doubt, the symptom was considered absent.

Twelve symptom characteristics were evaluated, including 8 characteristics known as typical of angina (precordial pain, compressive aspect, irradiation to the left upper limb, irradiation to the neck, intensity classified by the patient as severe, discomfort on the previous days, presence of vagal symptoms, administration of sublingual medication followed by improvement of the symptom) and 4 characteristics considered atypical (change of pain according to the position, change with palpation of the site, change with movement of the arm and change with breathing).

### Symptom typicality index

In order to quantify the overall typicality of the clinical manifestation, 1 point was assigned to each typical characteristic and 1 point was subtracted for each atypical characteristic (variation from -4 to +8, proportional to typicality).

### Definition of symptom etiology

For the diagnostic evaluation, the patients were submitted to invasive coronary angiography or non-invasive provocative test (nuclear perfusion magnetic resonance imaging and single-photon emission computed tomography or dobutamine stress echocardiography), at the discretion of the attending cardiologist. For positive noninvasive tests, the patients had an angiography done for confirmation. Based on this diagnostic algorithm, obstructive coronary artery disease (OCAD) was defined as stenosis ≥70% at angiography. Coronary angiography without obstructive lesion or normal noninvasive test (ischemic defect size <5% of left ventricular myocardium) indicated the absence of OCAD.

### Data analysis

Normality of numeric variables was tested by histogram, comparing mean and median, and mainly considering the level of kurtosis and skewness <3. Very elderly individuals were defined as age ≥80 years (octogenarians). Primary analysis was performed on the sample of patients with obstructive coronary disease, comparing the typicality index between octogenarians and non-octogenarians. In addition, each symptom characteristic was compared between the two groups. Numerical variables were expressed as mean and standard deviation, compared between the two groups by Student’s unpaired t-test. Categorical variables were expressed in proportions and compared using Pearson’s chi-squared test. Analysis of variance was done to compare the typicality index between groups after adjustment for gender. A linear association between typicality index and age was tested by Pearson’s correlation coefficient, based on a normal distribution of both variables. Multiple comparison was adjusted using the Bonferroni method.

Then, the total sample of the registry (all patients admitted with acute chest pain, with and without coronary artery disease) was used and we evaluated the predictive capacity of the typicality index for obstructive coronary artery disease based on the area under the Receiver Operator Characteristic (ROC) curve. Next, we evaluated the age-modifying effect on the diagnostic accuracy (OCAD) of the overall pain typicality, in terms of interaction vs. age typicality in logistic regression, with age being inserted in three different ways: as a numerical variable, categorized into two groups (octogenarians or non-octogenarians) and categorized into two groups from the sample median. The software SPSS Version 23 was used. Statistical significance was defined as a two-tailed p value smaller than 0.05.

### Sample size calculation

As for the sample size calculation, this was a study conducted on a previously existing sample as part of the Chest Pain Registry, a prospective collection of patients hospitalized for chest pain. This registry is used for various analyses and, in our methodology, before deciding to test any hypothesis, we evaluated the statistical power, which depends on the behavior of the variable in question. Thus, as the data had already been collected, we could use the standard deviation of the sample that would be used to evaluated whether the sample size was powered enough, an essential criterion to allow data analysis in our protocol.

Thus, sample size was defined first, based on the distribution of the typicality index in the coronary disease sample. Considering a standard deviation of 1.7, it would be necessary for 36 octogenarians and 109 non-octogenarians to offer 80% power in detecting a 30% difference in the typical index by the Student’s t test.

## Results

### Sample characteristics

Between September 2011 and December 2017, 958 individuals were included in the registry, and 486 (51%) had a supposedly coronary etiology. In this group, 59 octogenarians were compared to 427 non-octogenarians. The mean age of the octogenarians was 85±3.4 years, including 56% men, compared to 60±12 years, including 71% men, in the non-octogenarian group (p<0.001). Octogenarian patients had a higher prevalence of clinically manifested left ventricular dysfunction (24% versus 8.7%, p<0.001), triple vessel disease or left main coronary artery (41% versus 26%, p=0.01) and a lower prevalence of ST segment elevation acute myocardial infarction (25% versus 30%, p<0.001). Mortality was higher in the older group (14% versus 2.1%, p<0.001). The variables compared between the two groups are described in Table 1 .

**Table 1:** – Clinical characteristics and comorbidities

	Age <80 (n=427)	Age ≥80 (n=59)	p value
Age (years)	60±12	85±3.4	<0.001
Male	302 (71%)	33 (56%)	0.02
ECG ischemia	279 (67%)	37 (65%)	0.80
Positive troponin	274 (65%)	49 (83%)	0.005
ST-segment elevation infarction	127 (30%)	15 (25%)	<0.001
Diabetes mellitus	161 (38%)	27 (46%)	0.23
Creatinine (mg/dl)	1.0±0.69	1.1±0.43	0.12
Systolic blood pressure (mmHg)	154±31	153±36	0.08
Heart rate (bpm)	78±18	76±19	0.17
Previous coronary disease	139 (33%)	24 (41%)	0.22
Previous myocardial revascularization	37 (8.7%)	7 (12%)	0.40
Severe anatomical pattern*	80 (26%)	16 (41%)	0.01

*Catheterism with obstruction ≥70%; ECG: electrocardiogram.

### Age and typicality of symptoms

The typicality index of very elderly patients was 3.42±1.92, which is similar to that observed in younger individuals (3.44±1.74; p=0.92). Comparison of the typicality index remained non-significant (p=0.80) after adjusting for the gender difference between the groups ( Figure 1 ).

**Figure 1:** – Boxplot of the typicality index for the octogenarian and non-octogenarian groups (p=0.92).

There was no difference in the typicality index when the sample was divided into median age (62 years), being 3.41±1.77 versus 3.49±1.77 (p=0.61). Likewise, there was no correlation between typical index and age (r=- 0.05, p=0.24) ( Figure 2 ).

**Figure 2:** – Scatter plot of typical index and age.

The comparison between the 12 pain characteristics between octogenarians and non-octogenarians showed no significant difference after Bonferroni adjustment ( Table 2 ).

**Table 2:** – Characteristics of thoracic pain

	Age ≥80	Age <80	p value	Adjusted p value (Bonferroni)
Precordial pain	348 (82%)	48 (81%)	0.98	--
Compressive	241 (56%)	28 (48%)	0.19	--
Irradiation to upper left limb	167 (39%)	19 (32%)	0.02	0.24
Irradiation to the neck	110 (26%)	8 (14%)	0.04	0.48
Severe intensity	253 (60%)	39 (66%)	0.33	--
Discomfort on previous days	67 (48%)	14 (67%)	0.10	--
Vagal symptoms	215 (50%)	32 (54%)	0.58	--
Improvement with nitrate	182 (43%)	18 (31%)	0.08	--
Changes with position	70 (16%)	7 (12%)	0.37	--
Changes with palpation	26 (6.1%)	2 (3.4%)	0.40	--
Changes with arm movement	29 (6.8%)	3 (5.1%)	0.62	--
Pleuritic pain	51 (12%)	5 (8.5%)	0.43	--

### Age-modifying effect on predictive capacity of pain typicality

Analyzing the 958 patients in the registry, the typicality index presented an area under the ROC curve of 0.62 (95% CI = 0.58–0.65) for prediction of obstructive coronary artery disease. The logistic regression analysis showed no interaction of typicality index with numerical age (p=0.94), octogenarians (p=0.22) or age above the median of 62 years (p=0.74) ( Figure 3 ).

**Figure 3:** – C-statistics of typicality index for prediction of obstructive coronary artery disease, considering all patients in the registry (958), ROC curve: 0.62 (95% CI = 0.58–0.65).

## Discussion

The present study demonstrates that advanced age has no influence on the clinical presentation typicality in the context of acute coronary syndromes. In addition, the diagnostic value of the clinical manifestation is not influenced by age. As shown, even analyzing “old age” from various perspectives (dividing the sample between octogenarians and non-octogenarians, median age, 62 years, and still placing age as a continuous variable), none of the analyses suggested influence.

The use of a “typicality index” allowed us to analyze overall pain typology, information complemented by the individual analysis of each characteristic. The so-called “index” is just the count of suggested symptoms minus unsuggested symptoms, a way to treat typicality as a numerical variable, avoiding the subjectivity of categorization in a typical or atypical clinical picture.

Another important point of this study is that, for the definition of OCAD, we used coronary angiography, the gold standard exam, which implies a low risk of calibration bias.

Previous studies that sought to study pain in older individuals with acute coronary syndrome showed controversial results.^2 , 3 , 5^ It is found that in most of these studies the collection of pain characteristics was done retrospectively and from databases developed with other primary objectives.

In 2001, Mehta et al.,^1^ through a registry of Medicare beneficiaries in the USA, selected patients diagnosed of acute myocardial infarction and stratified the sample on the basis of age.^1^ In their study, the authors conclude that the initial presentation of chest pain decreased as the age increased. However, they do not show whether there is a statistical difference between the values, which makes this conclusion misleading.

In a post-hoc analysis of the Internet Tracking Registry for Acute Coronary Syndromes (i*trACS), Han et al.^3^ analyzed the clinical presentation in patients with ACS from two groups: age ≥75 years and <75 years. They classified “typical presentation” as thoracic pain in crushing, compression or pressure, and concluded that only in the group of younger patients (age <75 years) the typical presentation was associated with the diagnosis of ACS. In addition to the simplistic definition of “typical presentation,” the authors did not compare the two age groups diagnosed with ACS. In another post-hoc analysis, from the Gulf Registry of Acute Coronary Events (Gulf RACE), El-Menyar et al.^5^ classified into three categories: typical, atypical and dyspnea, and found no age difference in the “typical” (55±12) and “atypical” (57 ± 13) presentation groups. However, the authors attribute rather broad characteristics as being “typical”: “irradiation to the arm, shoulder, back, neck, jaw, epigastrium or other sites” which makes this classification subjective.

We must recognize that despite satisfying the sample size calculation, our population of very elderly patients was small. In addition, our study was carried out in only one center and in a selected population, thus, the development of new studies in this context is necessary. We also recognize that this study was carried out in a tertiary hospital environment, so we must be careful when extrapolating its results to the primary care environment. Our population of greatest interest is that of patients admitted to the coronary care unit, a population where the challenge of diagnostic discrimination is greater, because there is greater homogeneity of symptoms (gray zone of probability). This being our target population, there was no selection bias. Finally, there is a plethora of possibilities and combinations of symptoms to be included in an analysis such as this. But here, we are not trying to create a predictor score for the etiology of pain; we are just comparing the very elderly and not very elderly regarding the “typicality load.” Regardless of whether we contemplate all possible symptoms, the hypothesis test for the “typicality load” is not compromised. We are just assessing whether there is a symptom gradient between these two groups.

## Conclusion

In patients with chest pain of coronary etiology, advanced age does not seem to influence the typical clinical presentation, suggesting that symptoms should be interpreted regardless of age.
